# Low isavuconazole trough levels in critically ill patients with and without extracorporeal membrane oxygenation

**DOI:** 10.1128/aac.00577-25

**Published:** 2025-07-23

**Authors:** Rolf Erlebach, Alix Buhlmann, Rea Andermatt, Mattia M. Müller, Reto Schuepbach, Silvio D. Brugger, Sascha David, Daniel A. Hofmaenner

**Affiliations:** 1 Institute of Intensive Care Medicine, University Hospital Zurich 27243 , Zurich, Switzerland; 2 Department of Infectious Diseases and Hospital Epidemiology, University Hospital Zurich, University of Zurich 27243 , Zurich, Switzerland; 3 Department of Nephrology & Hypertension, Medical School Hannover 9177 , Hanover, Germany; University Children's Hospital Münster, Münster, Germany

**Keywords:** antifungals, therapeutic drug monitoring, pharmacokinetics, drug dosing, aspergillosis

## Abstract

Data on isavuconazole exposure in critically ill patients and particularly during extracorporeal membrane oxygenation (ECMO) are scarce, and therapeutic drug monitoring is not routinely performed. Critically ill patients admitted to a tertiary ECMO referral center from October 2017 to August 2024 with documented isavuconazole trough levels were retrospectively analyzed. First, measured isavuconazole trough blood levels and the occurrence of dose adjustments were analyzed in patients with and without ECMO support. Fifty-three adult patients were included, of whom 11 (21%) patients were on ECMO support at the first isavuconazole trough level measurement. Median isavuconazole trough level was overall 1.4 (interquartile range [IQR] 0.9–2.5) mg/L and did not differ between ECMO (1.3 [IQR 0.9–1.5] mg/L) and non-ECMO patients (1.6 [IQR 0.9–2.8] mg/L, *P* = 0.423). During the entire intensive care unit stay, individual doses were increased in 12 (23%) patients, of whom 5 were on ECMO support, whereas dosage was reduced or interrupted in 2 (4%) patients (both without ECMO support). Dose adjustments occurred irregularly and inconsistently after therapeutic drug monitoring, i.e., only in 6 (11%) patients after the initial therapeutic drug monitoring despite 37 (70%) drug levels being outside the target range of 2–4 mg/L. In conclusion, below targeted isavuconazole trough levels were common in critically ill patients investigated, but ECMO did not seem to have an additional negative influence. Dose adjustments appeared more frequently than previously reported, albeit irregularly performed. Regular therapeutic drug monitoring and protocolized dose adjustments should be investigated in future studies.

## INTRODUCTION

Isavuconazole (IVZ) (1), a triazole broad-spectrum antifungal agent, is a first-line treatment option of invasive (pulmonary) aspergillosis (2). Owing to the high mortality of the condition in critically ill patients (3, 4), sufficient blood trough levels of IVZ should ideally be achieved early and consistently (5). Therapeutic drug monitoring (TDM) may help to detect patients with low and high drug exposure and facilitate dosing adjustment accordingly. However, TDM seems to be used inconsistently for IVZ. Early pharmacologic investigations suggested predictable, linear pharmacokinetics (6), and clinical observations found similar blood levels compared to clinical trials (7–9). Thus, current guidelines on the management of aspergillosis suggest the use of TDM with IVZ only in specific settings, such as unresponsiveness to treatment, unexpected toxicity, drug-drug interactions, or during treatment of pathogens with elevated minimal inhibitory concentrations (2). Recent studies question this approach, showing higher variability and frequently low IVZ trough concentrations (IVZ C_trough_), especially in the critically ill patients and during extracorporeal therapies (10–14). This is in line with another azole (i.e., voriconazole) that is well known for serum trough level alterations in the context of extracorporeal membrane oxygenation (ECMO). In addition to this knowledge, it has also been reported that ECMO patients have *per se* an increased risk for invasive fungal disease (15, 16). Adequate drug dosing in this population is generally challenging (17, 18) due to changes in volume of distribution (VOD) and drug sequestration within the extracorporeal circuit (19, 20). IVZ, in its active form, is highly lipophilic and protein bound, with an inherent theoretical high risk of sequestration to the ECMO circuitry (19) (in analogy to reports on voriconazole). Evidence regarding TDM of IVZ during ECMO support is scarce and revealed inconsistent results (10, 21–24). Given that IVZ TDM is performed quite frequently at our center, we conducted this observational study of IVZ TDM in ECMO patients. We hypothesize based on our own experience that IVZ trough levels are lower during ECMO support, and increased doses are frequently applied. Our aim is to characterize IVZ levels in critically ill patients with and without ECMO support and to assess potential divergence from current dose recommendations due to inadequate drug exposure. We further aim to elucidate which clinical decisions were triggered by measured IVZ levels.

## MATERIALS AND METHODS

### Design and study population

This retrospective cohort study aimed to assess IVZ C_trough_ in adult critically ill patients with and without ECMO. All patients admitted to the intensive care unit (ICU) of the University Hospital Zurich, Switzerland, who were treated with IVZ since the drug approval by the Swiss Agency for Therapeutic Products (Swissmedic) in October 2017 were screened for inclusion up to August 2024. Inclusion criteria were age ≥18 years and any documented TDM of IVZ during ICU stay at minimum 2 days after the first dose of IVZ, which corresponds to the loading dose. Documented written or oral refusal for study participation led to exclusion.

### Institutional policies regarding IVZ dosing

IVZ was administered to treat suspected or proven *Aspergillus* infections or less frequently other invasive mold infections. The recommended dosage at our institution consists of a loading dose of 200 mg IVZ (corresponding to 372 mg of isavuconazonium sulfate) every 8 hours for 48 hours (6 × 200 mg for 2 days), followed by a maintenance dose of 1 × 200 mg IVZ daily (25). Intravenous and orally administered doses were considered equivalent. There is no recommendation for routine therapeutic drug monitoring at our institution. TDM was therefore performed if deemed appropriate by the treating physician, e.g., in patients at high risk of variable drug exposure, such as patients with ECMO support, large shifts in VOD (sepsis or kidney replacement therapy), renal or liver impairment, high clinical necessity of achieving sufficient levels or expected prolonged therapy, and may have changed over time. IVZ blood concentrations were determined 15 minutes before the next IVZ dose was administered (labeled as IVZ C_trough_). At our institution, a clear therapeutic range is not defined, but our in-house laboratory expects trough levels of 3–4 mg/L after more than 7 days of therapy to be a useful target range (26).

### Variables and data acquisition

Data were extracted from the in-house clinical information system and the patient data and monitoring system. Demographics and population characteristics at ICU admission, organ support, and indication for IVZ therapy were collected. IVZ doses, IVZ C_trough_, and duration of ECMO support were assessed. ICU length of stay and ICU mortality were registered. Invasive pulmonary aspergillosis (IPA) as an indication for IVZ was categorized according to the European Organisation for Research and Treatment of Cancer (EORTC; [27]) and invasive fungal diseases in adult patients in intensive care unit (FUNDICU; [15]) consensus definitions. Patients with suspected IPA who did not meet these criteria were classified as “other.” For immunosuppression, we used the definition of the Acute Physiology And Chronic Health Evaluation (APACHE) II score (28) and defined high-dose steroid therapy as a prednisone-equivalent dose of ≥7.5 mg/day. Obesity was defined as body mass index (BMI) ≥30 kg/m^2^.

### Statistical analysis

Population characteristics are presented for the entire cohort and stratified by ECMO status at the first measurement of IVZ C_trough_. Comparisons of IVZ C_trough_ based on ECMO support and between subgroups were limited to the first TDM to address bias introduced by multiple testing of the same individual. For the analysis of IVZ C_trough_, ECMO status was only defined as “yes” if ECMO support was currently applied at the time of IVZ measurement. IVZ C_trough_ was further categorized into four groups: insufficient (<1 mg/L), low (1–1.9 mg/L), target (2–4 mg/L), and high (>4 mg/L), based on existing guidelines (29) and the European Committee on Antimicrobial Susceptibility Testing (EUCAST) breakpoints (25). The target range was extended compared to the in-house laboratory target (3–4 mg/L after more than 7 days of therapy) because TDM in critically ill patients is frequently performed before 7 days of therapy, and clinicians may prefer to adhere to these guidelines (25, 29). Subgroups were chosen based on previous limited evidence for differences in pharmacokinetics based on sex, age, BMI, sequential organ failure assessment (SOFA) score (10, 11), and kidney replacement therapy (10, 14, 24). To examine therapeutic implications of TDM for IVZ, dose adjustments were quantitatively analyzed and visualized longitudinally in a flow chart, stratified by IVZ C_trough_. An individual patient summary is provided.

Variable distributions were assessed by visual inspection of histograms and quantile-quantile plots. Values are given as means with standard deviations, medians with interquartile ranges (IQRs), or counts with percentages, as appropriate. Medians were compared with the Wilcoxon rank sum test. Statistical analysis was performed through a fully scripted data management pathway using the R environment for statistical computing version 4.4.0. A two-sided *P* < 0.05 was considered statistically significant.

## RESULTS

### Population characteristics

Fifty-three patients who received IVZ during their ICU stay could be included in the analysis. ECMO support was applied in 21 (40%) of these patients and in 11 (21%) patients during the first measurement of IVZ C_trough_. The selection process and reasons for exclusion are shown in Fig. S1 as a study flow chart.

Median age in the study population was 56 (IQR 46–63) years, where 47% of patients were female. Patients had an initial SOFA score of median 10 (IQR 8–13) and a Charlson Comorbidity Index of median 3 (IQR 2–4). Forty-four patients (83%) were considered immunosuppressed. Solid organ transplantation state was registered in 16 (30%), and hematologic disease was present in 15 (28%) patients. The remaining 13 immunosuppressed patients were categorized, owing to their medication such as chronic steroid use. Indication of IVZ therapy was possible, probable, or proven IPA in 47 (89%) patients, and 10 (19%) patients received IVZ already before ICU admission for that reason. Population characteristics stratified by ECMO support at initial TDM are presented in Table 1.

**TABLE 1:** Population characteristics at ICU admission, ICU therapy, and overall outcomes stratified by ECMO support at initial IVZ C_trough_ measurement^*a*^

		ECMO status at initial TDM		
Characteristic	Overall (*N =* 53)	No (*N =* 42)	Yes (*N =* 11)	*P*-value
Age (years)	56 (46–63)	57 (51–63)	44 (23–61)	0.036^*b*^
Female sex	25 (47%)	18 (43%)	7 (64%)	0.219^*c*^
Body mass index (kg/m^2^)	26 (22–29)	26 (22–29)	27 (22–31)	0.561^*b*^
Scores				
SAPS II	52 (39–62)	53 (42–63)	44 (31–57)	0.160^*b*^
SOFA score	10 (8–13)	11 (9–13)	9 (7–11)	0.234^*b*^
Charlson Comorbidity Index	3 (2–4)	3 (2–5)	2 (0–3)	0.003^*b*^
Comorbidities				
Cardiovascular disease	15 (28%)	14 (33%)	1 (9.1%)	0.149^*d*^
Cerebrovascular disease	3 (5.7%)	3 (7.1%)	0 (0%)	>0.999^*d*^
Chronic obstructive pulmonary disease	6 (11%)	5 (12%)	1 (9.1%)	>0.999^*d*^
Chronic kidney disease (stages 3–5)	8 (15%)	8 (19%)	0 (0%)	0.181^*d*^
Liver disease	6 (11%)	4 (9.5%)	2 (18%)	0.592^*d*^
Diabetes mellitus	9 (17%)	7 (17%)	2 (18%)	>0.999^*d*^
Solid tumor	5 (9.4%)	5 (12%)	0 (0%)	0.571^*d*^
Solid organ transplantation	16 (30%)	14 (33%)	2 (18%)	0.471^*d*^
Hematologic disease	15 (28%)	14 (33%)	1 (9.1%)	0.149^*d*^
Immunosuppression	44 (83%)	36 (86%)	8 (73%)	0.372^*d*^
Host factors				0.591^*d*^
- EORTC	38 (72%)	31 (74%)	7 (64%)	
- FUNDICU	12 (23%)	9 (21%)	3 (27%)	
- None	3 (5.7%)	2 (4.8%)	1 (9.1%)	
Isavuconazole indication				0.330^*d*^
- Possible IPA	16 (30%)	14 (33%)	4 (36%)	
- Probable IPA	25 (47%)	20 (48%)	5 (45%)	
- Proven IPA	6 (11%)	6 (14%)	0 (0%)	
- Other	6 (11%)	3 (7%)	3 (27%)	
Isavuconazole before ICU admission	10 (19%)	9 (21%)	1 (9.1%)	0.667^*d*^
ICU therapy				
Mechanical ventilation	49 (92%)	38 (90%)	11 (100%)	0.569^*d*^
Kidney replacement therapy	31 (58%)	29 (69%)	2 (18%)	0.004^*d*^
ECMO canulation (n=21)				0.183^*d*^
- Venovenous ECMO	14 (67%)	5 (50%)	9 (82%)	
- Venoarterial ECMO	7 (33%)	5 (50%)	2 (18%)	
Outcomes				
ICU length of stay (days)	34 (20–55)	30 (17–47)	52 (29–73)	0.017^*b*^
ICU mortality	23 (43%)	16 (38%)	7 (64%)	0.177*^d^*

^*a*^ Values are presented as median (interquartile range) or count (%). SAPS, simplified acute physiology score.

^*b*^ Wilcoxon rank sum test.

^*c*^ Pearson’s chi-squared test.

^*d*^ Fisher’s exact test—no adjustment for multiple testing.

### Isavuconazole blood levels with and without ECMO

In total, 127 IVZ C_trough_ levels were measured, corresponding to an average of 2 (range 1–10) measurements per patient. The first IVZ C_trough_ drawn at median 6.1 (IQR 4.0–11.9) days after the first administered IVZ dose in the ICU was further analyzed. At this time point, 11 (21%) patients were supported by ECMO. Median IVZ C_trough_ was 1.4 (IQR 0.9–2.5) mg/L and did not differ based on ECMO status (*P* = 0.423). Insufficient IVZ C_trough_ (<1 mg/L) was observed in 3 (27%) cases with ECMO and 13 (31%) cases without ECMO (Table 2). Distribution of IVZ C_trough_ grouped by ECMO status is shown in Fig. S2.

**TABLE 2:** First IVZ C_trough_ stratified by ECMO status^*a*^

		ECMO		
Characteristic	Overall (*N =* 53)	No (*N =* 42)	Yes (*N =* 11)	*P*-value
Median IVZ C_trough_	1.4 (0.9–2.5)	1.6 (0.9–2.8)	1.3 (0.9–1.5)	0.423^*b*^
Grouped IVZ C_trough_				0.4^*c*^
- Insufficient (<1 mg/L)	16 (30%)	13 (31%)	3 (27%)	
- Low (1–1.9 mg/L)	17 (32%)	11 (26%)	6 (55%)	
- Target (2–4 mg/L)	16 (30%)	14 (33%)	2 (18%)	
- High (>4 mg/L)	4 (8%)	4 (10%)	0 (0%)	

^*a*^ Values are presented as median (interquartile range) or count (%).

^*b*^ Wilcoxon rank sum test.

^*c*^ Fisher's exact test.

### Subgroup analysis

**Fig 1:**
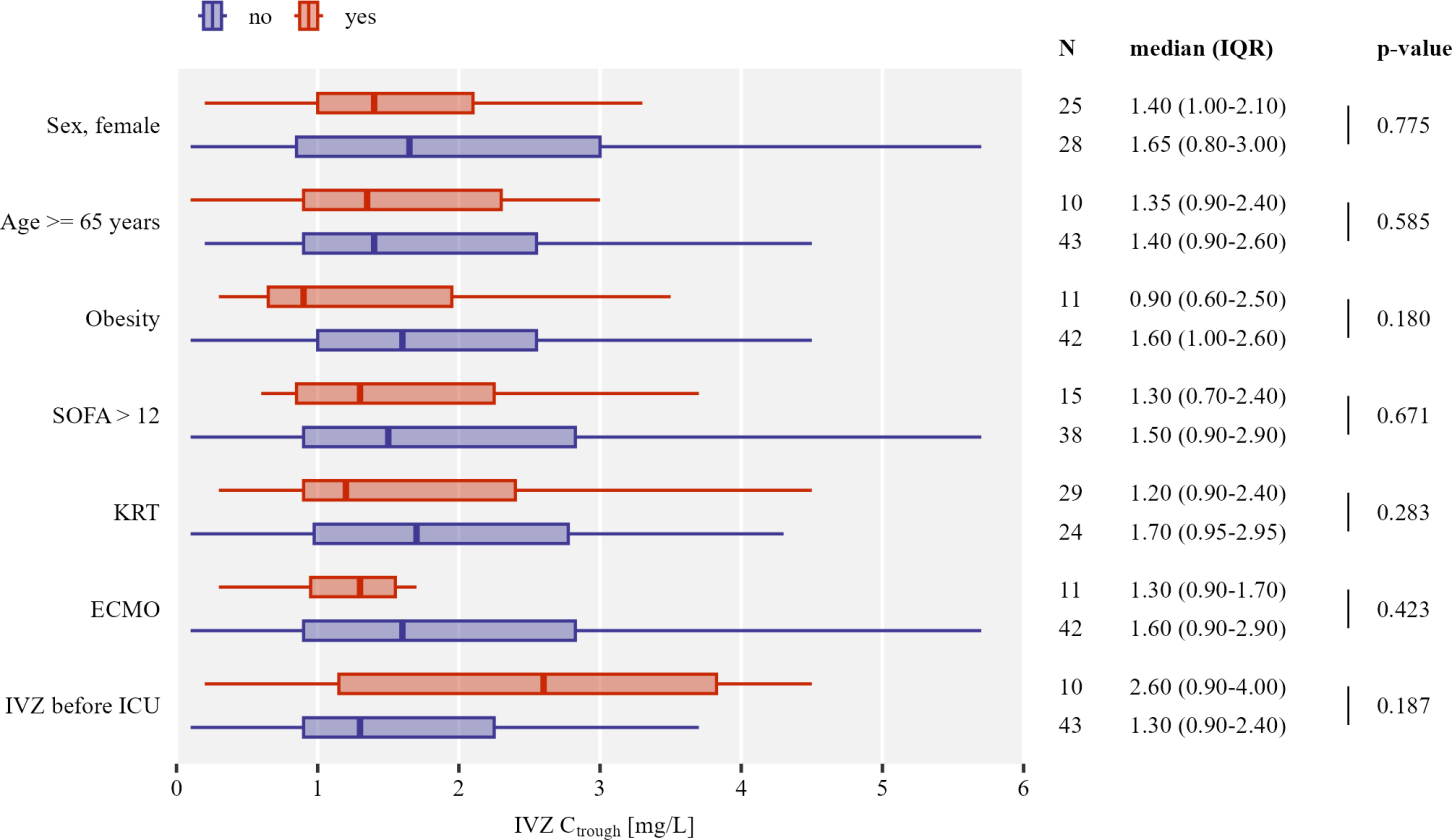
Horizontal boxplot chart of IVZ C_trough_ (first measurement per patient) grouped by subgroups. Outliers are not displayed for better visualization. Numerical results of the number of observations, central tendency (median [IQR]), and *P*-value for the Wilcoxon rank sum test are displayed on the right side of the plot. KRT, kidney replacement therapy; Obesity, body mass index >30 kg/m^2^.

Subgroup analysis based on sex, age, obesity, SOFA score, kidney replacement therapy, ECMO, and IVZ therapy before ICU admission did not reveal any differences in median IVZ C_trough_ between these subgroups (Fig. 1).

### Dose adjustments over time

**Fig 2:**
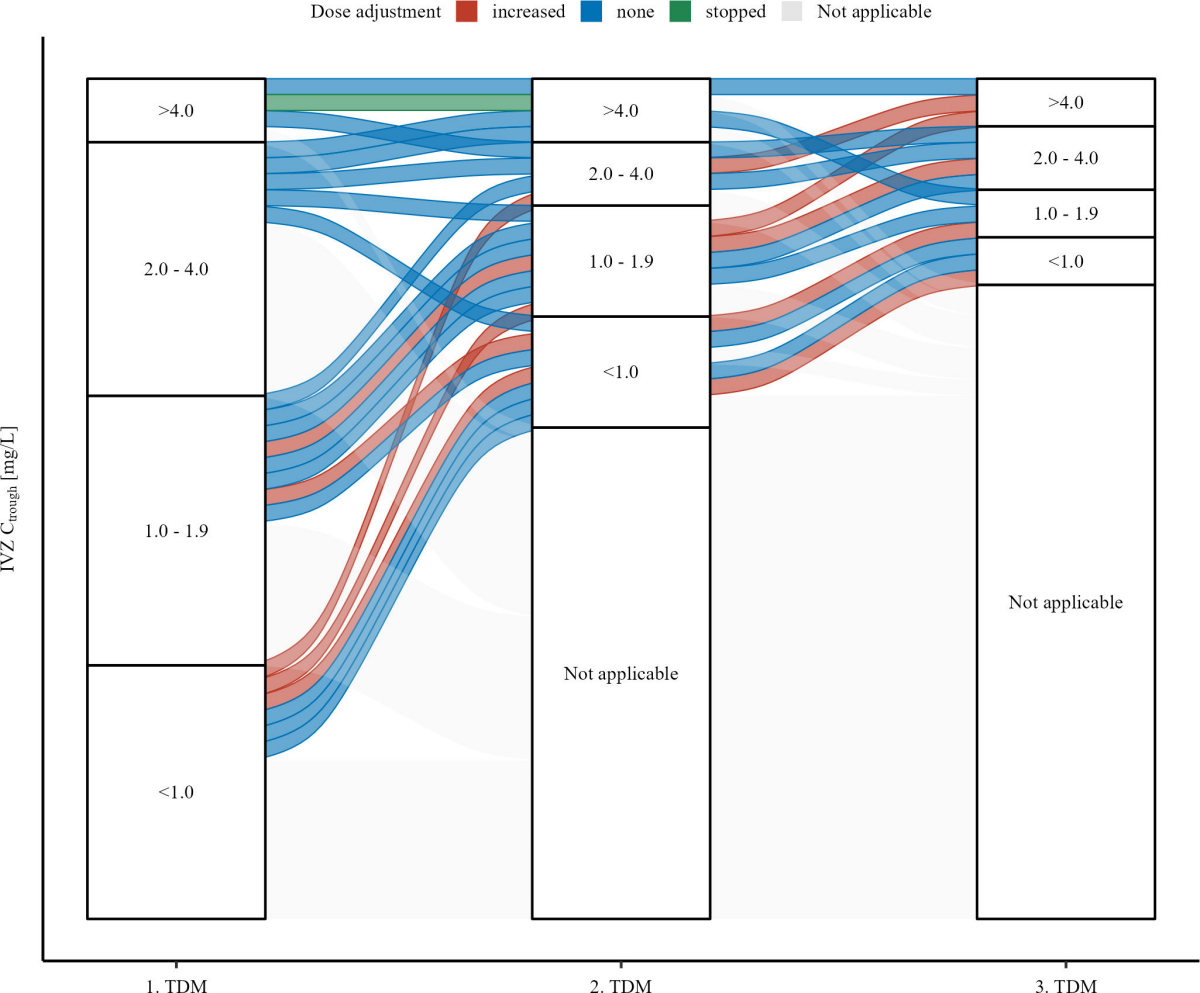
Flow chart of dose adjustment between TDM measurements. Each flow represents one patient. Colors represent dose adjustments made between measurements (increased, stopped/reduced, or unchanged). Patients who did not have a subsequent TDM are colored in light gray (“Not applicable”). Strata represent categories of IVZ C_trough_ measurements (<1.0, 1.0–1.9, 2.0–4.0, or >4.0 mg/L) or “Not applicable” if no subsequent measurement was performed.

During the entire ICU stay, individual doses were increased in 12 (23%) patients, of whom 5 were on ECMO support, compared to 7 without ECMO at time of measurement. IVZ dosage was interrupted or reduced in two (4%) patients (both without ECMO support). Considering only the first measurement of IVZ C_trough_, in 3 (19%) patients with insufficient levels and in 2 (12%) patients with low levels, the IVZ dose was increased immediately (before a next measurement or ICU discharge/death). Dose reduction/interruption was observed in one (25%) patient with a high level at the first measurement. Dose adjustments occurred irregularly and inconsistently after TDM (Fig. 2) despite 37 (70%) IVZ levels being outside the target range of 2–4 mg/L. Interestingly, in the majority of observed low IVZ levels, no dose increase was performed by the clinicians in charge (Fig. 2). A similar pattern with lacking dose adjustments was observed even after subsequent second IVZ measurements (Fig. 2). Of note, some subsequent IVZ levels decreased in subsequent measurements despite the dose having been increased (Fig. 2).

### Individual patient treatment tracks

For all included patients, individual details of IVZ doses, TDM, ICU therapies over time, and referral status are summarized in Fig. S3.

## DISCUSSION

In this retrospective observational study assessing real-life IVZ levels in critically ill patients with and without ECMO support, TDM of IVZ revealed low (<2 mg/L) and insufficient IVZ C_trough_ (≤1 mg/L) in a substantial part of patients (30% and 32%). Median IVZ doses did not differ between measurements during ECMO and measurements obtained without ECMO. Surprisingly, administered doses were only increased in 19% of patients with insufficient levels and in 12% patients with low levels. IVZ dosage was reduced or interrupted only in one patient. Interestingly, clinicians frequently did not increase IVZ dosage despite the measurement of low IVZ C_trough_.

Currently, there is no international consensus about the ideal target range for IVZ C_trough_ levels, in general, and particularly not in critically ill patients with or without organ support. Nevertheless, a minimal efficacy target for IVZ C_trough_ of 1 mg/L has been proposed by some authors (21, 22) in order to achieve blood levels above the EUCAST clinical breakpoint for sensitivity of *Aspergillus fumigatus* and *A*. *flavus* (25). This is also supported by the fact that the epidemiological cut-off values for *A. fumigatus* and *A*. *flavus* have been reported as being 2 mg/L (>2 mg/L also reflects the resistance breakpoint) (25). However, in clinical practice, an IVZ C_trough_ of >2 (–4) mg/L might often be more helpful to match the mean exposure attained in the phase III SECURE trial (1, 2, 23). Even a higher target of >2.5 mg/L has been suggested during prolonged IVZ therapy (30). A target range of 2–4 mg/L has also been suggested by the Dutch Working Party on Antibiotic Policy (29), but this suggestion is based on modeling studies only and combined with the recommendation to measure IVZ trough levels first after 3 days and weekly afterward until the target has been reached.

In our critically ill cohort, a substantial part of patients with and without ECMO exhibited IVZ blood levels below the minimal threshold of 1 mg/L, and the median IVZ C_trough_ was considerably low.

Our findings of low IVZ exposure in critically ill patients are also consistent with those of a recent study by Ergün et al., in which individual IVZ area under the curve measurements over 7 days exceeded the target threshold of 60 mg·h/L in only fewer than 40% of patients. This underscores the high prevalence of IVZ underdosing in critically ill patients, in general, and highlights the need for individualized and longitudinal monitoring of IVZ drug exposure (31).

The distribution of IVZ levels showed more frequent high IVZ levels in patients without ECMO. The results of this study are in concordance with other reports of IVZ blood levels in critically ill ECMO patients (10, 21, 23) and are in striking contrast with observations in non-critically ill patients, where blood levels of >1 mg/L were found in >90% of patients (7, 32). Possible explanations for this difference include an increased VOD in ICU patients and higher clearance due to altered protein binding, in general (10). If ECMO with its circuit itself further increases the patient’s VOD to a clinically relevant amount, thus further altering IVZ blood levels is questionable (33, 34). Sequestration of highly protein-bound and lipophilic drugs by the ECMO circuit is another mechanism that has been described previously and was considered a major problem of voriconazole treatment during ECMO (17, 18, 35). For IVZ, the impact of drug sequestration, however, might be negligible, as pre- and post-oxygenator IVZ levels were shown to be unaffected by the oxygenator (34). Although not significant, ECMO patients in our cohort tended to have lower IVZ blood levels in line with existing studies (10, 22, 24), supporting the hypothesis that ECMO itself might further lower IVZ levels in critically ill patients. On the other hand, our observed lack of difference in IVZ levels between patients with and without ECMO support might, in fact, be a potential advantage of IVZ compared to voriconazole in patients needing ECMO support. Of note, no patient on ECMO support achieved high doses in our cohort. Future studies should also assess the impact of oxygenator changes on levels of IVZ during an ECMO run. Owing to too low numbers of circuit changes in our cohort, this analysis was currently not feasible.

Regarding dose adjustments, our study yielded interesting results with relevant clinical implications. While over 60% of patients had IVZ C_trough_ levels below previously suggested target ranges (2–4 mg/L) (29), which is even lower than our in-house target after 7 days (3–4 mg/L), dose increase at the first TDM was seldom performed. This contradicts previously published data from haemato-oncological pediatric patients, where dosage increases were needed in only 3% of cases for IVZ compared to 54% of cases for voriconazole (36). In our study, clinicians frequently did not increase IVZ doses despite measured low or insufficient IVZ C_trough_ levels, irrespective of the ECMO treatment status. A similar effect was observable when subsequent (i.e., second or third) IVZ C_trough_ were measured. Moreover, we observed that some IVZ levels were even lower in subsequent measurements compared to the initial measurements despite performed dose increases. These findings highlight the importance of drawing the right consequences at the bedside when TDM for IVZ is applied. Future prospective studies should directly assess whether TDM with integrated protocolized dose adjustments will translate into higher frequencies of sufficient IVZ levels or even improved patient outcomes in a clinical condition with often high morbidity and mortality. Other authors have shown that increasing the first loading dose of IVZ leads to faster attainment of IVZ C_trough_ >1 mg/L in ECMO patients (22). IVZ blood levels may further differ based on the time of exposure. Increasing IVZ blood levels over time have been reported before (30). While this effect is also important to consider in non-ICU settings, such as prolonged outpatient treatment or prophylaxis, particularly in the ICU setting, sufficient blood levels should ideally be achieved after the first dose, owing to the severity of patient disease. Delayed sufficient therapy could be detrimental in the setting of invasive aspergillosis, as shown in an experimental model with immunosuppressed mice (5).

Our predefined subanalyses did not reveal different IVZ levels in particular subgroups. Kidney replacement therapy, which is frequently applied in ECMO patients, has been linked to lower IVZ blood levels (10, 14, 24). This association could not be reproduced by our data. The analysis was, however, limited by a small number of patients with kidney replacement therapy. Other factors that have been associated with lower IVZ blood levels are female sex, SOFA score >12, obesity, and older age (10, 11). We also found lower IVZ C_trough_ in females and in obese patients; however, it was not statistically significant. Owing to limited patient numbers, these measurements should be considered exploratory and might be hypothesis generating for further studies.

In general, future research should aim to implement clearly defined prospective protocols, including standardized IVZ sampling time points, during ECMO support and predefined strategies for dose adjustments when drug levels fall outside the target range. In addition, studies are needed to determine whether specific dosing recommendations can be established for clinical situations in which ECMO is initiated or discontinued, considering potential alterations in pharmacokinetics directly linked to the ECMO device itself. The influence of ECMO parameters (such as blood flow rates) on IVZ pharmacokinetics also warrants further assessment. Furthermore, it remains important to evaluate whether TDM of IVZ, applied through standardized protocols, is directly linked to improved clinical outcomes, including infection control, morbidity, and mortality.

Our analysis has several strengths, including real-world data from 53 critically ill patients with IVZ treatment. TDM in this population is exceptionally important to expand the limited knowledge of drug dosing in the ICU and particularly during ECMO support. Our results suggest that standard dosing regimens may not be sufficient in critically ill patients and that increased doses may be effective in achieving higher blood levels without exceeding a proposed threshold for toxicity. This has high clinical relevance with practical consequences.

This study also has some limitations, including its single-centric and retrospective design.

A causal relationship between IVZ C_trough_ and patients or treatment characteristics cannot be drawn. As ECMO is a life support strategy of last resort, the number of patients with IVZ treatment was rather low and potentially heterogeneous. Besides the heterogeneity of patients, the time period of ECMO, as a temporary support strategy, is also highly individual and may not correlate with the initiation of IVZ therapy and TDM. Thus, we did not compute multivariable mixed effect models. Moreover, we did not differentiate between the protein bound and the free, pharmacologically active fraction of IVZ, which might limit the interpretability of our IVZ levels to some extent. This is supported by previous data demonstrating high variability in IVZ protein binding, particularly in critically ill patients (37). Future research thus should directly assess the effects of critical illness and ECMO therapy on the free IVZ fraction more in depth.

In future studies, higher sample sizes and a multicenter approach should ideally be chosen to adjust for confounders. As TDM was not standardized, C_trough_ measurements were performed after different durations of therapy, which might impede comparison between patients. Nonetheless, our data provide important insights into the drug levels of IVZ in the ICU and show that not only monitoring but standardized dose adjustment protocols may be substantial to avoid insufficient drug exposure.

### Conclusion

IVZ blood levels below 1 mg/L under standard dosing measured at a median of 6 days after IVZ initiation occurred in about one-third of patients with and without ECMO. Adjustments are more frequently necessary than previously reported, albeit irregularly performed in real life by clinicians. Regular TDM and protocolized dose adjustments should be investigated in future studies.

## Data Availability

For this retrospective non-experimental study, ethics regulations do not allow sharing of health-related data from patients involved. Upon reasonable request, exemption will be sought from the local ethics commission.

## SUPPLEMENTAL MATERIAL

Supplemental material aac.00577-25-s0001.pdf Fig. S1 to S3.

ASM does not own the copyrights to Supplemental Material that may be linked to, or accessed through, an article. The authors have granted ASM a non-exclusive, world-wide license to publish the Supplemental Material files. Please contact the corresponding author directly for reuse.
