# Supplementary material for: Low isavuconazole trough levels in critically ill patients with and without extracorporeal membrane oxygenation
Source: Antimicrob Agents Chemother. 2025 Jul 23;69(9):e00577-25. doi: 10.1128/aac.00577-25 (PMC12406667; doi:10.1128/aac.00577-25)
Supplement: Supplemental material — Fig. S1 to S3. [file aac.00577-25-s0001.pdf]

Supplemental Material

Low Isavuconazole trough levels in critically ill patients with and without extracorporeal membrane oxygenation

Rolf Erlebach, Alix Buhlmann, Rea Andermatt, Mattia M. Müller, Reto Schuepbach, Silvio D. Brugger, Sascha David, Daniel A. Hofmaenner

Figure S1

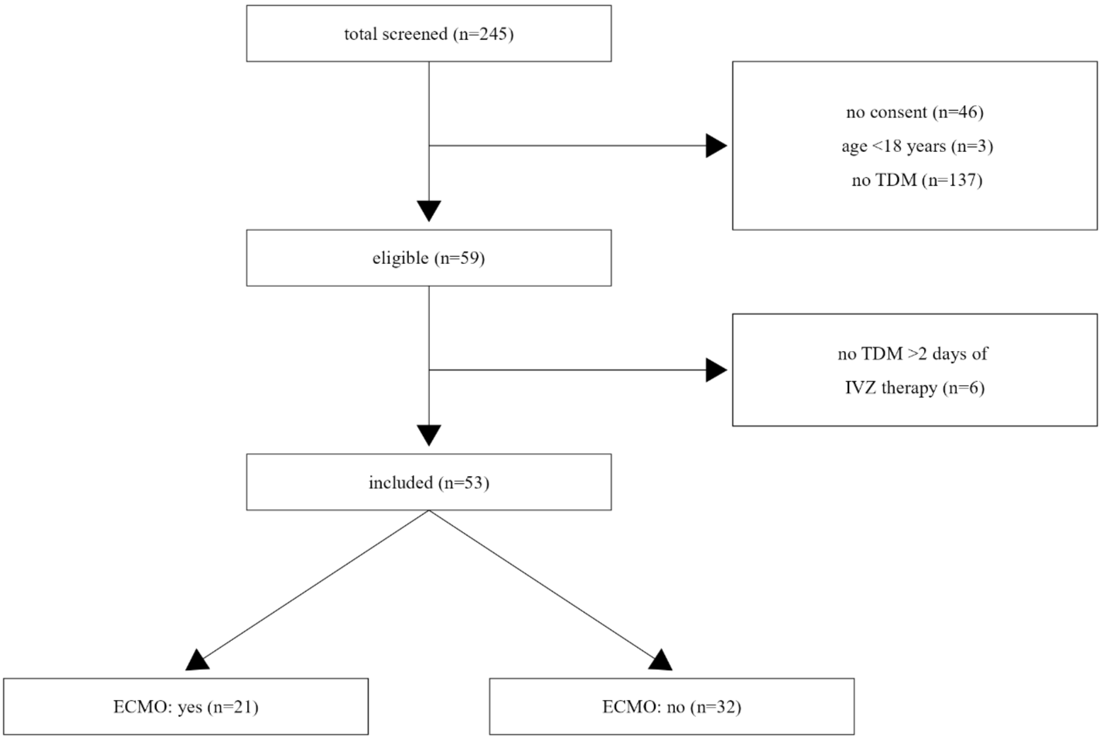

Figure 1: Flow chart of screening and inclusion of patients. ECMO: Extracorporeal Membrane Oxygenation, IVZ: Isavuconazole, TDM: Therapeutic drug monitoring.

Figure S2

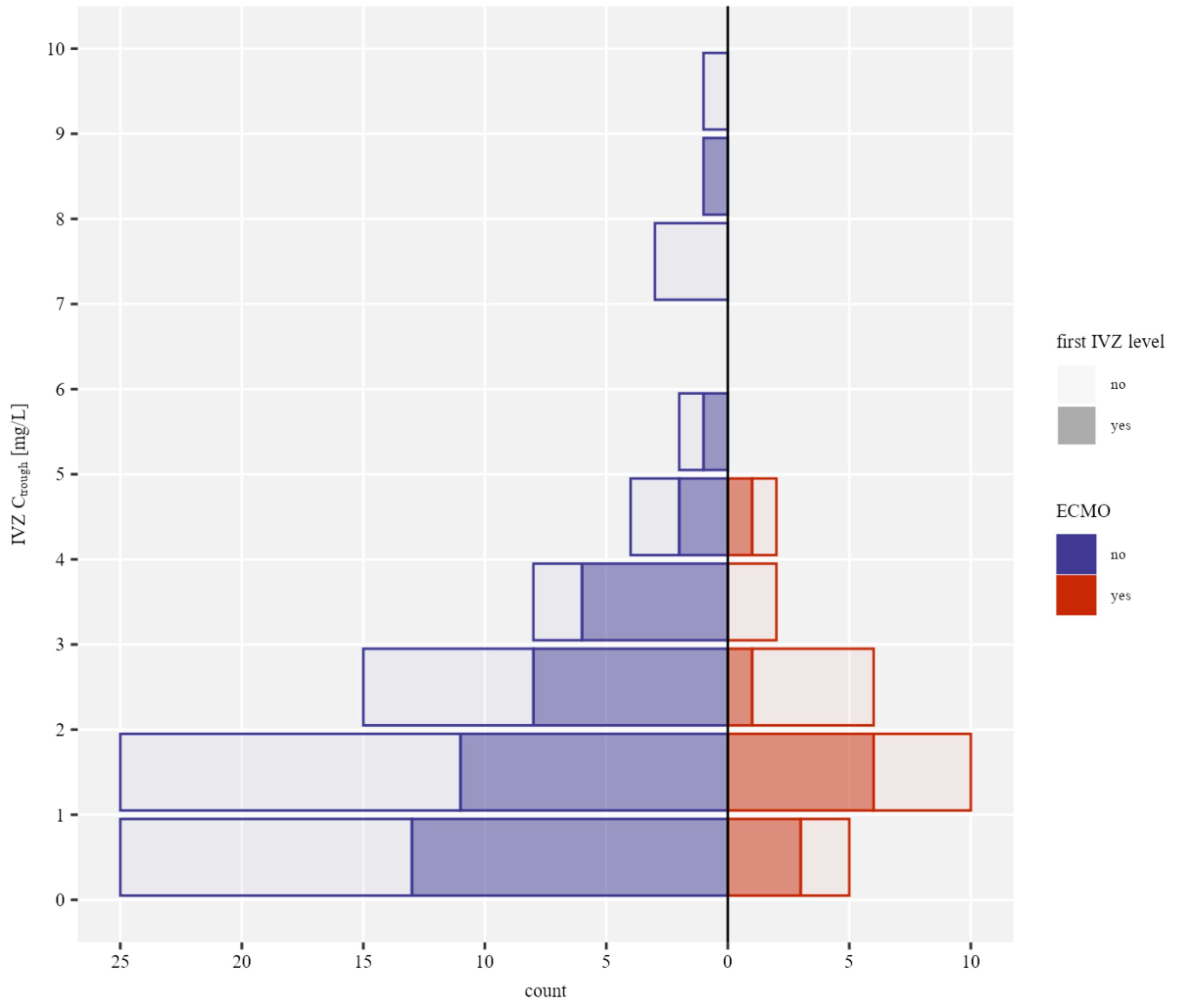

Figure S2: Horizontal bar chart of IVZ C_{trough} grouped by ECMO status (red vs. blue color). The transparent or shaded colors indicate whether the IVZ C_{trough} was the first measured trough level per patient or not (e.g. a subsequent measurement). IVZ C_{trough}: Isavuconazole trough blood concentration, ECMO: Extracorporeal membrane oxygenation

Figure S3

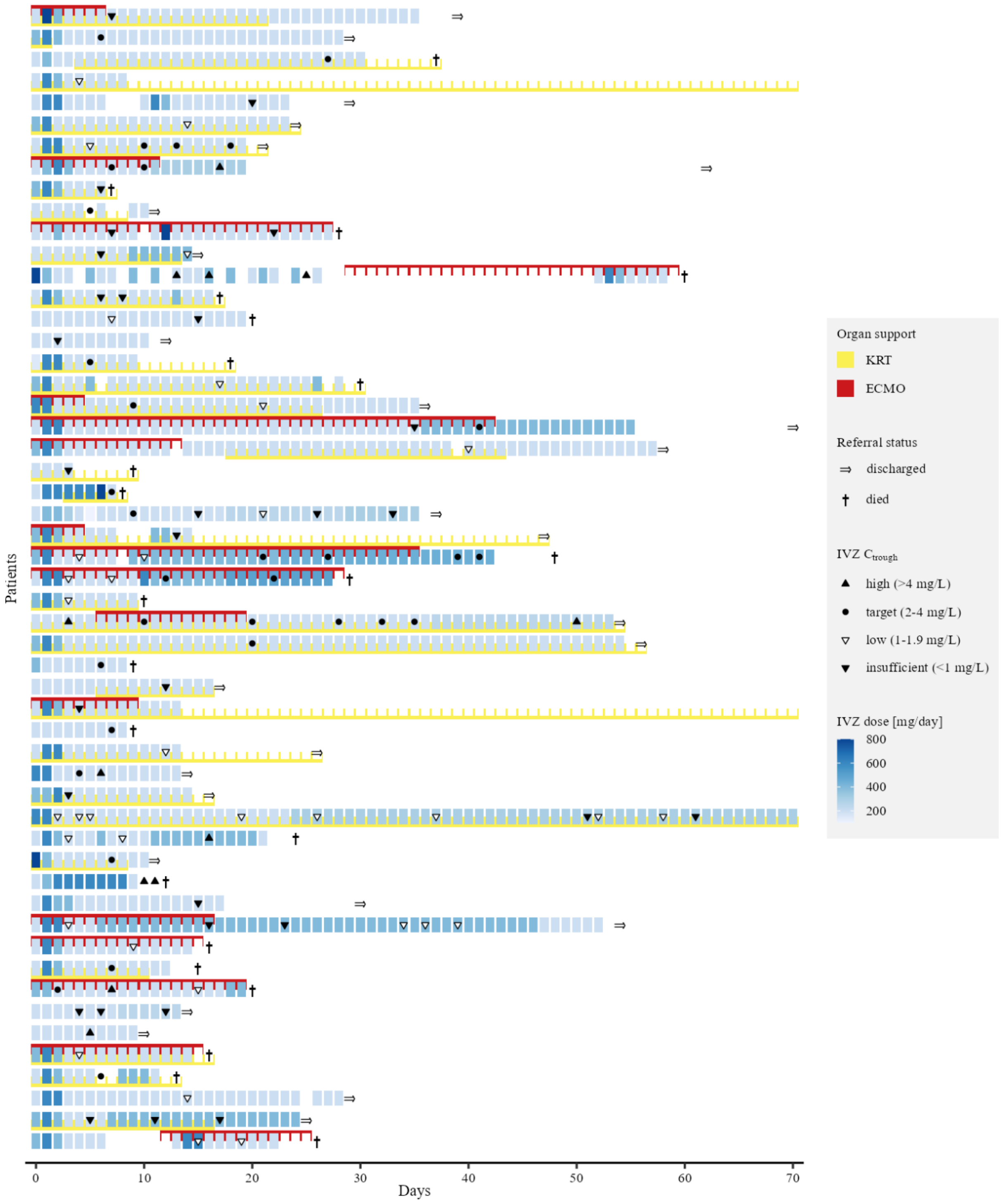

Figure S3: Individual patient summary chart starting from the first dose of IVZ until discharge or death and truncated at 70 days. Each row represents one patient. The x-axis represents days from the first dose of IVZ administered at the intensive care unit. Daily IVZ dose is calculated continuously starting from the first dose in 24 hours intervals. ECMO: Extracorporeal membrane oxygenation, KRT: Kidney replacement therapy, IVZ: Isavuconazole.
